# Supplementary material for: Antibiotic treatment does not abrogate protection against Mycobacterium tuberculosis reinfection in Macaques
Source: bioRxiv. 2023 Dec 19:2023.12.19.570845. Preprint. [Version 1] doi: 10.1101/2023.12.19.570845 (PMC10769216; doi:10.1101/2023.12.19.570845)
Supplement: Supplementary file 1 [file media-3.docx]

**Supplementary Figures:**

**Figure S1. Total lung PET-CT FDG activity reflects inflammatory changes over the course of the experiment.** Serial FDG PET CT scans were performed throughout the primary and secondary challenge. Total lung FDG activity is shown for the Abx (A) and the two no-Abx (B, C) groups. Purple shaded area indicates duration of drug treatment and pink shaded area reflects the time and duration of the secondary challenge with Mtb.
